# Tumor Necrosis Factor-α Induces a Preeclamptic-like Phenotype in Placental Villi via Sphingosine Kinase 1 Activation

**DOI:** 10.3390/ijms23073750

**Published:** 2022-03-29

**Authors:** Yuliya Fakhr, Saloni Koshti, Yasaman Bahojb Habibyan, Kirsten Webster, Denise G. Hemmings

**Affiliations:** 1 Department of Obstetrics and Gynecology, University of Alberta, Edmonton, AB T5H 3V9, Canada; fakhr@ualberta.ca (Y.F.); koshti@ualberta.ca (S.K.); bahojbha@ualberta.ca (Y.B.H.); kawebste@ualberta.ca (K.W.); 2 Women and Children’s Health Research Institute, Edmonton, AB T6G 1C9, Canada; 3 Department of Microbiology and Immunology, University of Alberta, Edmonton, AB T6G 2E1, Canada

**Keywords:** sphingolipids, inflammatory, sphingosine 1-phosphate, syncytium

## Abstract

Preeclampsia (PE) involves inadequate placental function. This can occur due to elevated pro-inflammatory tumor necrosis factor-α (TNF-α). In other tissues, TNF-α signals via sphingosine kinase 1 (SphK1). SphK1 hinders syncytial formation. Whether this occurs downstream of TNF-α signaling is unclear. We hypothesized that placental SphK1 levels are higher in PE and elevated TNF-α decreases syncytial function, increases syncytial shedding, and increases cytokine/factor release via SphK1 activity. Term placental biopsies were analyzed for SphK1 using immunofluorescence and qRT-PCR. Term placental explants were treated after 4 days of culture, at the start of syncytial regeneration, with TNF-α and/or SphK1 inhibitors, PF-543. Syncytialization was assessed by measuring fusion and chorionic gonadotropin release. Cell death and shedding were measured by lactate dehydrogenase release and placental alkaline phosphatase-positive shed particles. Forty-two cytokines were measured using multiplex assays. Placental SphK1 was increased in PE. Increased cell death, shedding, interferon-α2, IFN-γ-induced protein 10, fibroblast growth factor 2, and platelet-derived growth factor-AA release induced by TNF-α were reversed upon SphK1 inhibition. TNF-α increased the release of 26 cytokines independently of SphK1. TNF-α decreased IL-10 release and inhibiting SphK1 reversed this effect. Inhibiting SphK1 alone decreased TNF-α release. Hence, SphK1 partially mediates the TNF-α-induced PE placental phenotype, primarily through cell damage, shedding, and specific cytokine release.

## 1. Introduction

Preeclampsia (PE) is a pregnancy disorder characterized by high blood pressure and end organ damage. PE is the cause of 76,000 maternal and 500,000 annual deaths globally and increases the rate of preterm and low birthweight neonates [1,2]. The cause of PE remains elusive, albeit the placenta plays a major role in its initiation and progression.

The human placenta is a complex organ composed of multiple cell types performing different functions and interacting with each other’s signaling pathways. Placental tissue includes cells found in other organs such as endothelial cells, vascular smooth muscle cells, fibroblasts, and immune cells. However, the unique cell type of the placenta is the pluripotent cytotrophoblast (CT) that can differentiate into an extravillous trophoblast lineage, responsible for anchoring the placenta into the maternal decidua, or into the villous trophoblast lineage [3].

The villous CT proliferates, differentiates, and then fuses into the basal side of the overlying syncytiotrophoblast (ST), a multinucleated epithelial layer that regulates feto-maternal exchanges. The ST also acts as an immune and pathogen barrier between mother and fetus. This placental layer is in direct contact with maternal blood and releases hormones, such as human chorionic gonadotropin (hCG), essential for maintaining the pregnancy [3].

Similar to other epithelial cells, the ST is in constant turnover throughout pregnancy, due to a balance between formation of the ST through differentiation and fusion of CTs and loss of the ST through shedding. The mature ST aggregates into syncytial knots and sheds into the maternal bloodstream. These syncytial aggregates are characterized by high levels of placental alkaline phosphatase (PLAP) enzyme activity [3].

The placenta in PE is characterized by poor development of the syncytium, high levels of shedding and cell damage, and high levels of inflammatory cytokines [3]. In PE, low CT proliferation leads to impaired differentiation and reduced fusion to ST, disrupting syncytial formation and thus, function. Abnormally high levels of placental cell death and ST shedding in PE [4] lead to a variety of pro-inflammatory responses [5] which further exacerbate the PE phenotype [6]. 

Pro-inflammatory cytokines are notorious for disrupting placental function, notably tumor necrosis factor-α (TNF-α) [7]. Elevated TNF-α levels are linked to pregnancy complications including recurrent spontaneous abortions [8], preterm labor [9], and PE [10]. Injecting TNF-α alone into pregnant non-human primates leads to the development of PE symptoms [11]. Moreover, elevated TNF-α causes a PE-like placental phenotype that includes poor ST function, high ST shedding, and high inflammation [12,13].

Physiological levels of TNF-α, however, have pregnancy enhancing roles such as inducing mucin 1 shedding from uterine epithelial cells, a major feature of embryo implantation [14]. Normal levels of TNF-α also regulate the fate [15,16], proliferation [17,18,19], and differentiation [20,21] of various cells, including CTs [7,12]. TNF-α maintains normal levels of apoptosis that are necessary for fetal development [22]. This makes market-available TNF-α inhibitors controversial for use during pregnancy [23]. Our aim was to explore alternative factors downstream of TNF-α signaling that can be targeted. 

One of the mediators of TNF-α signaling in non-placental endothelial and epithelial cells is sphingosine 1-phosphate (S1P) [24,25,26,27]. S1P is a bioactive sphingolipid that regulates cellular processes such as angiogenesis, proliferation, differentiation, and apoptosis [28]. S1P acts intracellularly, or it can exert its effects extracellularly through its specific receptors, S1P receptors 1–5 (S1PR1-5), which are variably expressed in all placental cell types and have multiple functions as summarized in Fakhr et al. [28]. Briefly, S1PR1 and S1PR3 activation in trophoblasts leads to the secretion of IL-8. In the placental vasculature, S1PR1 activation in endothelial cells leads to filament reorganization that is necessary for conserving the endothelial barrier and this receptor is also involved in vasodilation. Moreover, S1PR1 activation in the placental vasculature decreases reactive oxygen species and pro-inflammatory cytokine release. S1PR2 activation decreases trophoblast migration that is important for attaching the placenta to the maternal decidua. S1PR4 and S1PR5 are mainly expressed in placental cells of immune origin [28].

S1P is synthesized by sphingosine kinases 1/2 (SphK1, SphK2) and is degraded by S1P lyase (SPL) or dephosphorylated by S1P phosphatases (SPP) and lipid phosphate phosphatases (LPPs) [28]. SphK1 activation and S1P are implicated downstream of TNF-α signaling [29]. In endothelial cells, the TNF-α signaling pathway involves S1P and SphK1 as cofactors for TNF-α receptor 1 (TNFR1) activation [24,30,31]. The addition of TNF-α to HUVECs [25,26], hepatocytes [32], or a lung fibroblast cell line [33] also activates SphK1.

S1P is also associated with inflammatory pathologies like atherosclerosis [34], cancer [35], and multiple sclerosis [36]. Circulating levels of S1P are higher in women with PE [37,38], where inflammation plays a role. High levels of S1P inhibit syncytialization [39] and inhibiting SphK1 increases trophoblast fusion [40]. Although the individual roles that TNF-α and S1P play in the pathogenesis of PE have been studied, we do not know whether TNF-α mediates its effects in the placenta through S1P by affecting SphK1 expression or activity. Given the importance of both TNF-α and S1P on placental function and pregnancy outcomes, it is essential to understand the interaction between TNF-α and SphK1, the main producer of extracellular S1P, in the placenta. We hypothesized that high levels of TNF-α would decrease syncytialization, increase shedding, and increase placental cytokine release through activation of SphK1. We show that TNF-α activated SphK1 to induce placental damage, ST shedding, and cytokine release. However, at lower concentrations, TNF-α decreased CT fusion independently of SphK1 activation.

## 2. Results

### 2.1. SphK1 Expression in Placentas from Normal and PE Pregnancies

Seventeen women with healthy pregnancies and 12 women with PE were enrolled in the study and their clinical characteristics are summarized in Table 1. Women with PE had higher systolic (155.3 ± 7.6 versus 113.8 ± 3.7 mmHg; *p* < 0.0001) and diastolic (107.9 ± 15.6 versus 71.4 ± 3.6; *p* = 0.008) blood pressures compared to those in the healthy control group. All participants in the PE group were positive for proteinuria. Infants born to mothers in the PE group had lower gestational ages (37.2 ± 0.3) compared to those from healthy control mothers (38.9 ± 0.3; *p* = 0.003). There were no significant differences in infant birth weights between groups.

**Figure 1:**
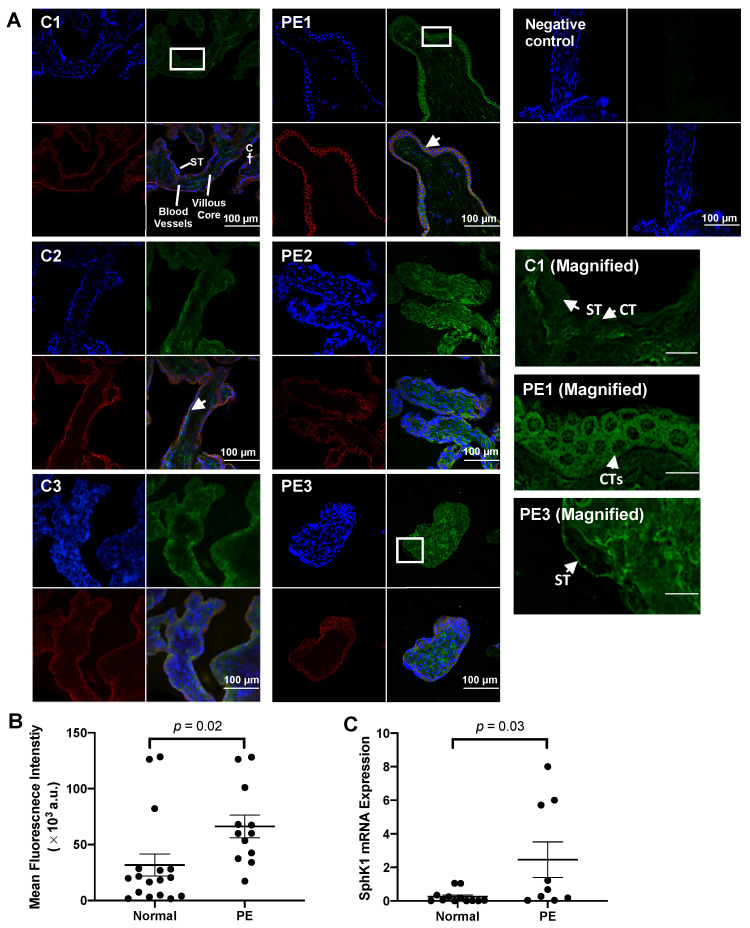
Sphingosine kinase 1 (SphK1) expression was higher in placental biopsies from women with preeclampsia (PE). (**A**): SphK1 was expressed throughout the villi, the cytotrophoblast (CT), and the syncytium (ST). Images correspond to three representative samples from healthy control pregnancies (C1, C2, C3 images) and PE (PE1, PE2, PE3 images). Whole tissue placental biopsies from women with normal or PE pregnancies were co-stained for SphK1 (AF488, green) and E-cadherin (AF594, red) expression and a nuclear stain (DAPI, blue) and visualized by confocal microscopy. For the negative control, both AF488 and AF594 secondary antibodies were used in the absence of primary antibodies. White arrows show examples of overlap between E-cadherin and SphK1 (**B**): Whole sample image analysis normalized to total nuclear count showed higher SphK1 fluorescence in placental biopsies from women with PE compared to healthy control pregnancies. Student’s *t*-test (*n* = 17 normal, *n* = 12 PE, Mean ± SEM). (**C**): Fold change in SphK1 mRNA expression in placental biopsies from women with PE relative to healthy control pregnancies using the ΔΔCt method. Student’s *t*-test (*n* = 13 normal, *n* = 9 PE, Mean ± SEM).

Previous studies show that dysregulated SphK1-S1P levels are observed in placentas from women with PE, and high levels of S1P or SphK1 activity are inhibitory to ST development [40]. We investigated whether cell-specific expression of SphK1 differed in placental biopsies from women with PE compared to those with normal pregnancies. To visualize expression differences in placental layers and cell types, we used immunohistochemistry. SphK1 was expressed throughout the villous core and in both CTs and the multinucleated ST, as shown in the magnified images C1, PE1, and PE3 (Figure 1A). In both the healthy control and PE groups, SphK1 expression rarely overlapped with the nuclear stain while SphK1 exhibited a strong overlap with the E-cadherin staining, as identified with white arrows. This implies that in trophoblastic layers, SphK1 is mainly localized to the cytoplasm and in close proximity to the cell membrane. Overall, no overt qualitative differences in SphK1 expression between the PE (*n* = 12) and the healthy control (*n* = 17) groups were observed in the trophoblastic layers (Figure 1A), but SphK1 expression appeared higher in the villous core of biopsies from the PE group, as shown in PE2 and PE3. Quantitative analysis of whole sample images demonstrated increased SphK1 overall in placental biopsies from women with PE (*p* = 0.02; Figure 1B). In Figure 1B, there were three samples in the healthy group that displayed much higher levels of SphK1 staining intensity. Upon comparing clinical characteristics of these women with the rest of the group, we did not see any differences in blood pressure, gestational age or infant birth weight, that would suggest a resemblance to those characteristics seen in a woman with PE. There were no differences by fetal sex either.

Quantitative analysis of SphK1 mRNA expression demonstrated increased SphK1 in placental biopsies from women with PE (*p* = 0.03; Figure 1C; *n* = 9). In Figure 1C, there were 3 samples in the PE group that displayed much higher levels of SphK1 mRNA expression. Upon comparing clinical characteristics of these samples with the rest of the PE group, we did not see any differences in systolic or diastolic blood pressure, gestational age, infant birth weight, or fetal sex. PE is a highly clinically heterogenous disorder that can be caused by distinct factors. Benton et al. report subtypes of PE based on placental gene expression clusters [41]. This heterogeneity and the various presentations of placental dysfunction in PE can explain the heterogeneity that we see in SphK1 expression, specifically the mRNA expression.

### 2.2. Experimental Villous Explant Model of Syncytial Self-Regeneration

PE develops after the second trimester of pregnancy [42]. Even though PE starts developing during the first trimester, the poor development and function of the placenta during the second trimester exacerbates the disorder to clinically detectable levels. During the second trimester, the ST is characterized by high levels of regeneration to form an intact placental barrier. In this study, we used a villous explant culture model to investigate the interaction of TNF-α and SphK1 and their effects on ST regeneration and function, cell death, and cytokine and factor release during the regenerative phase of the ST layer. 

**Figure 2:**
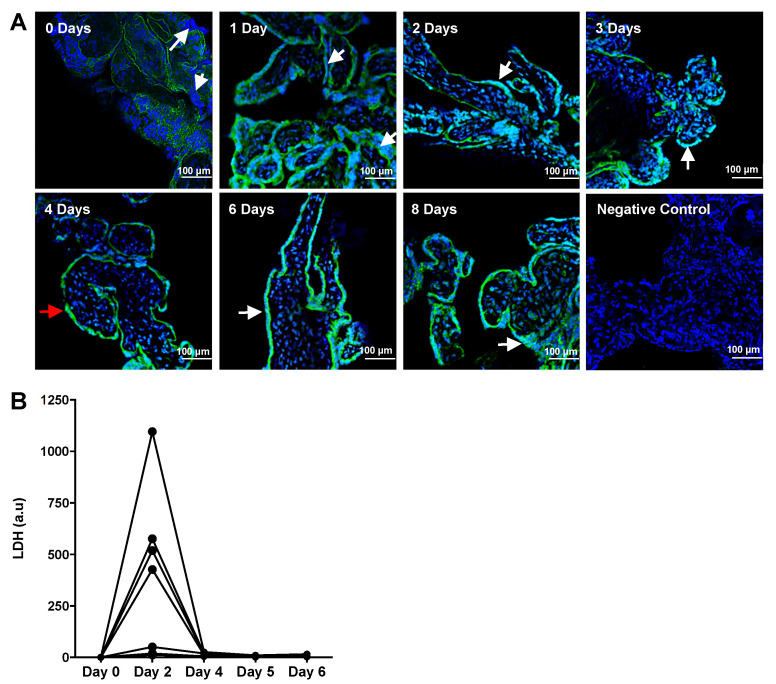
Syncytial shedding in chorionic villous explants was maximal after 4 days of incubation. (**A**): Confocal fluorescence microscopy was used to analyze the shedding of ST and resyncytialization of term chorionic explants after incubation for 0–8 days. DAPI stained nuclei blue and E-cadherin (AF488) stained epithelial cadherin junctions in CT and ST cell membranes green (*n* = 5). High E-cadherin expression demarcates the basal membrane of the syncytium. Nuclei present on the apical side of the heavy E-cadherin demarcation are part of the multinucleated ST. High areas of multinucleated syncytium are demarcated with white arrows and low areas of multinucleated syncytium are demarcated with red arrows. For the negative control, AF488 secondary antibodies were used in the absence of primary antibody. (**B**): Lactate Dehydrogenase (LDH) released into supernatants from chorionic explants was measured across various timepoints. Linked data points correspond to individual experiments (*n* = 6) and each data point represents the average of duplicate wells. Measurements correspond to LDH release in the last 24 h prior to every displayed time point. Values were plotted as a change from day 0. All values are relative to total protein mass in each culture well.

To characterize the time in culture needed for the ST to be fully shed prior to regeneration of this layer, explants were incubated for varying timepoints from dissection after delivery (0 days) up to 8 days and then assessed for the presence of ST using immunohistochemistry (Figure 2; *n* = 3). E-cadherin is expressed at epithelial adherens junctions, which are found in both CTs and the ST in the placenta, since trophoblasts are epithelial cells. High E-cadherin expression was saturated at the basal ST membrane where the apical side of the CT fuses into the ST. Since ST differentiation involves downregulating E-cadherin expression, the apical side of the ST expresses E-cadherin at low levels and is often not visualized with the microscope parameters optimal for the signal on the basal membrane of the ST. Any nuclei present on the apical side of the heavy E-cadherin demarcation belong to the multinucleated ST layer. Explants incubated for 0, 1, 6, or 8 days after initiation of culture showed multiple nuclear clusters on the apical side of the strong E-cadherin demarcation that define areas of multinucleated ST (Figure 2A, white arrows). Explants incubated for 2 or 3 days showed progressively less nuclei outside the E-cadherin staining than seen at 0 and 1 days, implying a reduced syncytial area. By day 4 of incubation, the ST was almost fully shed (Figure 2A, red arrow). Day 4 of culture was then used as the first day of treatment for all further experiments. Multiple areas of multinucleated ST reappeared on days 6–8 (Figure 2A, white arrows) of culture showing evidence for regeneration of the ST layer. LDH levels peaked at day 2 of culture and dropped again at day 4 of culture, showing evidence of ST sloughing before day 4 of culture (Figure 2B).

### 2.3. Effect of TNF-α on the Syncytium and its Interaction with SphK1

**Figure 3:**
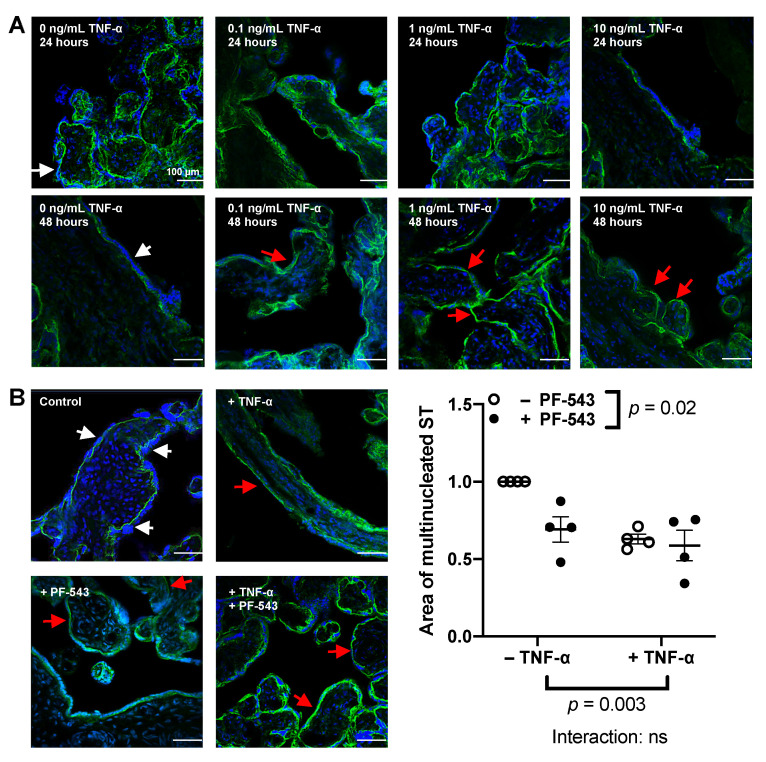
Resyncytialization in term placental chorionic explants from normal pregnancies decreased in response to tumor necrosis factor-alpha (TNF-α) and SphK1 +inhibition independently. (**A**): Explants were pre-cultured for 4 days prior to treatment to induce syncytial shedding. Resyncytialization of term chorionic explants following treatment with 0–10 ng/mL of TNF-α after 24 or 48 h of treatment was analyzed using confocal fluorescence microscopy. DAPI stained nuclei blue and E-cadherin (AF488) stained adherens junctions green (*n* = 3). (**B**): Explants were pre-cultured for 4 days prior to treatment with 1 ng/mL of TNF-α, 1 μM PF-543, a co-treatment of both or a no- treatment control for 48 h. Results were analyzed by measuring total area of the syncytium and normalizing it to the total surface area of the chorionic explant. Results are presented as relative to the no-treatment control and analyzed by two-way ANOVA with the two-stage linear step-up procedure of Benjamini, Krieger, and Yekutieli post-hoc test. (Mean ± SEM, *n* = 4).

Since SphK1 expression and activity is increased in response to proinflammatory stimuli [43,44] and S1P increases epithelial cell extrusion [45], we investigated the effects of SphK1 inhibition, using PF-543, on ST formation, function, and shedding in normal and proinflammatory conditions. We first identified the concentration of TNF-α that demonstrated maximum effects on resyncytialization in the explant model. After the initial 4-day culture to allow syncytial shedding, the explants were treated with TNF-α for 24 or 48 h (*n* = 3). As shown in Figure 3A, resyncytialization of term placental chorionic explants from normal pregnancies increased from 24 to 48 h in the no-treatment control. After 48 h of incubation without TNF-α, the syncytium was relatively thick and consistent around the villi (white arrow). There were no overt differences seen with TNF-α treatment after 24 h among the different TNF-α concentrations and the no-treatment control. However, 48 h of treatment with 0.1, 1, or 10 ng/mL of TNF-α resulted in reduced syncytium (red arrows). Treatment with 1 or 10 ng/mL of TNF-α for 48 h led to the most consistent and reproducible hinderance of resyncytialization compared to the no-treatment control, and hence those were the chosen concentrations for further experiments.

TNF-α treatment (1 ng/mL) decreased the formation of multinucleated ST area after 48 h (*p* = 0.003, *n* = 4, Figure 3B), regardless of PF-543 treatment. SphK1 inhibition with PF-543 (1 μM) also decreased the area of the multinucleated ST after 48 h (*p* = 0.02, *n* = 4, Figure 3B), regardless of TNF-α treatment. The area of multinucleated ST was not different after co-treatment of the explants with TNF-α and PF-543 when compared to individual TNF-α or PF-543 treatments (*n* = 4). We analyzed samples after 24 h of treatment as well, but there were no significant differences among the groups. Levels of β-hCG released into the supernatant were measured in explants treated with 1 ng/mL TNF-α, 1 µM PF-543, or a combined treatment of 1 ng/mL TNF-α and 1 μM PF-543 after 24 or 48 h of treatment. None of these treatments affected β-hCG (*n* = 6) or LDH (*n* = 8) levels released by the explants (Supplementary Figure S1). 

**Figure 4:**
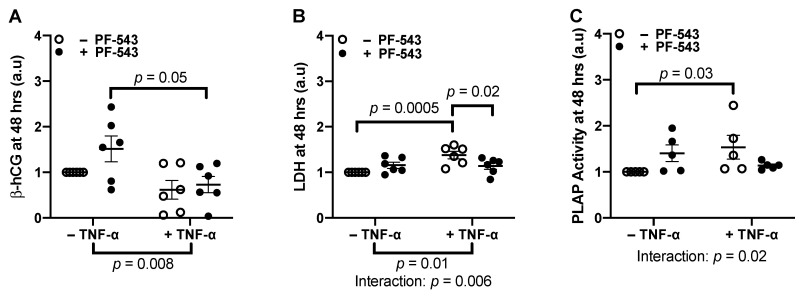
Inhibiting SphK1 with PF-543 diminished TNF-α effects on LDH release and placental alkaline phosphatase (PLAP)-positive shed particles but not on beta-human chorionic gonadotropin (β-hCG) secretion at 48 hrs. Explants were pre-cultured for 4 days prior to treatment with 10 ng/mL of TNF-α, 1 μM PF-543, a co-treatment of both or a no-treatment control for 48 h. Supernatants were centrifuged at 15,000 RPM for 15 min prior to analysis. (**A**) β-hCG released after 48 h was measured by ELISA. (**B**) LDH released into the supernatant after 48 h was measured with a colorimetric assay. (**C**): Non-centrifuged supernatants were analyzed for PLAP activity using an alkaline phosphatase activity assay. Results were normalized against total protein mass, and normalized values were graphed as a ratio of change from the untreated control explant cultures. Results were analyzed by two-way ANOVA with the two-stage linear step-up procedure of Benjamini, Krieger, and Yekutieli post-hoc test. (*n* = 6 or *n* = 5, Mean ± SEM).

Since 1 ng/mL TNF-α had no significant effects on β-hCG and LDH release, we tested whether a higher TNF-α concentration could hinder explant resyncytialization and increase cell death through SphK1. Explants were treated with 10 ng/mL TNF-α, 1 μM PF-543, or the combination of 10 ng/mL TNF-α and 1 µM PF-543 (*n* = 6). No significant changes were observed in β-hCG levels compared to controls in any of the groups at 24 h post-treatment (Supplementary Figure S2A). At 48 h post-treatment, however, 10 ng/mL TNF-α decreased β-hCG release (*p* = 0.008). However, inhibiting SphK1 (1 μM PF-543) did not counteract this decrease (Figure 4B). Furthermore, administration of 10 ng/mL TNF-α in explants with 1 μM PF-543 significantly decreased β-hCG levels to control levels compared to 1 μM PF-543 treatment alone (*p* = 0.05). This is likely due to the inhibitory effects of TNF-α as the treatment interaction was not significant. As well, β-hCG levels after co-treatment with PF-543 and TNF-α compared to TNF-α alone were not significant. This indicates that SphK1 activity does not mediate TNF-α effects on this ST endocrine function. 

No significant changes were observed in LDH levels compared to controls in any of the groups at 24 h post-treatment (Supplementary Figure S2B). However, TNF-α increased cell membrane damage as detected by increased LDH release at 48 h (10 ng/mL, *p* = 0.01, Figure 4B) that was inhibited by co-treatment with PF-543 (1 μM) with an interaction of *p* = 0.006. Post-hoc tests further confirmed this interaction as TNF-α increased LDH levels (*p* = 0.0005) and inhibiting SphK1 in the presence of TNF-α decreased LDH levels from TNF-α-induced levels (*p* = 0.02). Since the 24-h treatment showed no differences in LDH release, we measured ST shedding only at 48 h. ST shedding, demarcated by positive PLAP activity in the culture supernatant, was elevated in response to TNF-α treatment alone (10 ng/mL, *p* = 0.03, Figure 4C, *n* = 5). This effect was diminished upon Sphk1 inhibition (1 μM) with a treatment interaction of *p* = 0.02.

### 2.4. Effect of TNF-α on Placental Cytokine and Factor Release and Its Dependence on SphK1 Activity

**Figure 5:**
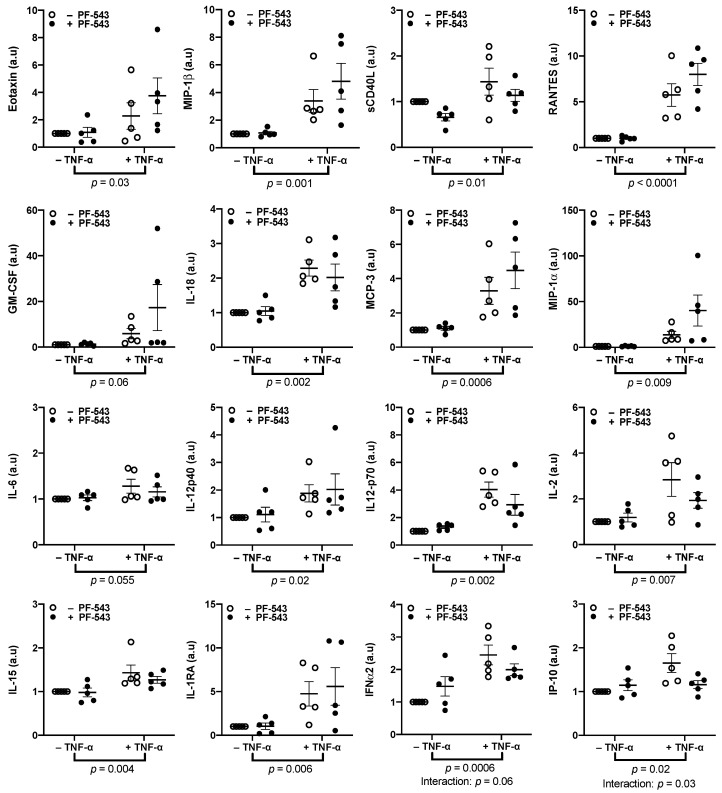
Inhibiting SphK1 with PF-543 diminished TNF-α effects on release of IFNα2 and IFN-γ associated protein 10 (IP-10). Explants were pre-cultured for 4 days prior to treatment with 10 ng/mL of TNF-α, 1 μM PF-543, a co-treatment of both, or a no-treatment control for 48 h. Supernatants were centrifuged at 15,000 RPM for 15 min prior to quantification of pro-inflammatory cytokines and chemokines using a multiplex array. Results were normalized against total protein mass, and normalized values were graphed as a ratio of change from the untreated control explant cultures. Results were analyzed by two-way ANOVA with the two-stage linear step-up procedure of Benjamini, Krieger, and Yekutieli post-hoc test. (*n* = 5, Mean ± SEM).

Treating explants with 10 ng/mL of TNF-α increased the release of the inflammatory cytokines eotaxin (*p* = 0.03) and MIP-1β (*p* = 0.001) after 48 h (Figure 5, *n* = 5). The treatment also increased the release of sCD40L (*p* = 0.01), RANTES (*p* < 0.0001), GM-CSF (*p* = 0.06), IL-18 (*p* = 0.002), MCP-3 (*p* = 0.0006), MIP-1α (*p* = 0.009), IL-6 (*p* = 0.055), IL-12p40 (*p* = 0.02), IL-12p70 (*p* = 0.002), IL-2 (*p* = 0.007), IL-15 (*p* = 0.004), and IL-1RA (*p* = 0.006), cytokines whose circulating levels are elevated in PE (Figure 5). The increased release of these factors in response to TNF-α was independent of SphK1 activation. TNF-α also increased the release of IFNα2 (*p* = 0.0006) and IFN-gamma-associated protein 10 or IP-10 (*p* = 0.02). The increase in these cytokines was reversed by SphK1 inhibition with a treatment interaction of *p* = 0.06 and *p* = 0.03, respectively (Figure 5).

**Figure 6:**
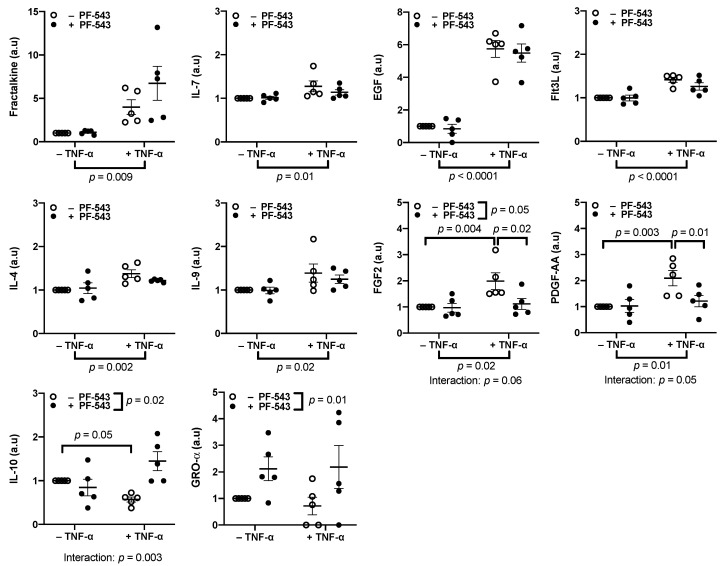
Inhibiting SphK1 with PF-543 diminished TNF-α effects on release of FGF2, PDGF-AA, and IL-10. Explants were pre-cultured for 4 days prior to treatment with 10 ng/mL of TNF-α, 1 μM PF-543, a co-treatment of both, or a no-treatment control, for 48 h. Supernatants were centrifuged at 15,000 RPM for 15 min prior to quantification of growth factors and chemokines using a multiplex array. Results were normalized against total protein mass, and normalized values were graphed as a ratio of change from the untreated control explant cultures. Results were analyzed by two-way ANOVA with the two-stage linear step-up procedure of Benjamini, Krieger, and Yekutieli post-hoc test. (*n* = 5, Mean ± SEM).

**Figure 7:**
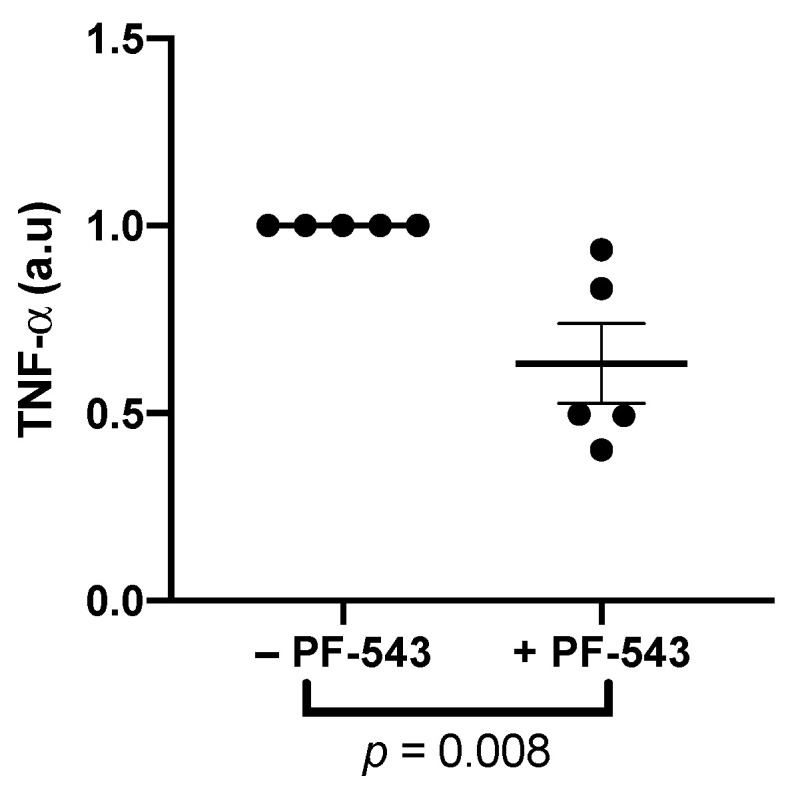
Inhibition of SphK1 decreased release of TNF-α. Explants were pre-cultured for 4 days prior to treatment with 1 μM PF-543 or a no-treatment control for 48 h. Supernatants were centrifuged at 15,000 RPM for 15 min prior to quantification of TNF-α using a multiplex array. Results were normalized against total protein mass, and normalized values were graphed as a ratio of change from the untreated control explant cultures. (*n* = 5, Mean ± SEM, Mann–Whitney test).

TNF-α also increased the release of the following growth factors (Figure 6, *n* = 5): fractalkine (*p* = 0.009), IL-7 (*p* = 0.01), EGF (*p* < 0.0001), and Flt3L (*p* < 0.0001). Two interleukins that are decreased in PE, IL-4 (*p* = 0.002) and IL-9 (*p* = 0.02), were also increased by this treatment. The increased release of these factors was independent of SphK1 activation. TNF-α increased the release of FGF2 (*p* = 0.02) and PDGF-AA (*p* = 0.01) (Figure 6) which was dependent on SphK1 since co-treatment of TNF-α with PF-543 decreased this release (*p* = 0.02 and *p* = 0.01, respectively) with treatment interactions of *p* = 0.06 and *p* = 0.05, respectively. Post-hoc tests further confirmed this interaction as TNF-α increased FGF2 (*p* = 0.004) and PDGF-AA (*p* = 0.003) and inhibiting SphK1 in the presence of TNF-α decreased FGF2 (*p* = 0.02) and PDGF-AA (*p* = 0.01) levels from TNF-α-induced levels. PF-543 treatment showed an overall change in FGF2 levels (*p* = 0.05). This change, however, was most likely due to its interaction with TNF-α, since post-hoc tests showed no difference between PF-543 treated groups and the no-treatment control. The only cytokine tested that was decreased by TNF-α treatment alone was IL-10 (*p* = 0.05). PF-543 treatment in combination with TNF-α not only reversed this decrease but further increased IL-10 release to 44.7 ± 20.6% higher than the no-treatment control with a treatment interaction of *p* = 0.003. This highlights the PF-543 treatment effect (*p* = 0.02). Inhibiting SphK1 with PF-543 increased GRO-α release (*p* = 0.01, Figure 6) but TNF-α had no effect on this cytokine. GRO-α was not detected in some samples and their levels were designated as 0 on the graph, and they were excluded from statistical analysis. Neither TNF-α or PF-543 treatments had any effect on IFNγ, IL-1-α, IL-1β, IL-5, IL-8, MCP-1, G-CSF, and PDGF-BB (Supplementary Figure S3). Inhibiting SphK1 in the absence of TNF-α treatment decreased TNF-α release (*p* = 0.008, Figure 7). TNF-α and GRO-α were the only factors that were altered in response to PF-543 treatment alone.

### 2.5. PF-543 Dose Response in Villous Explants

**Figure 8:**
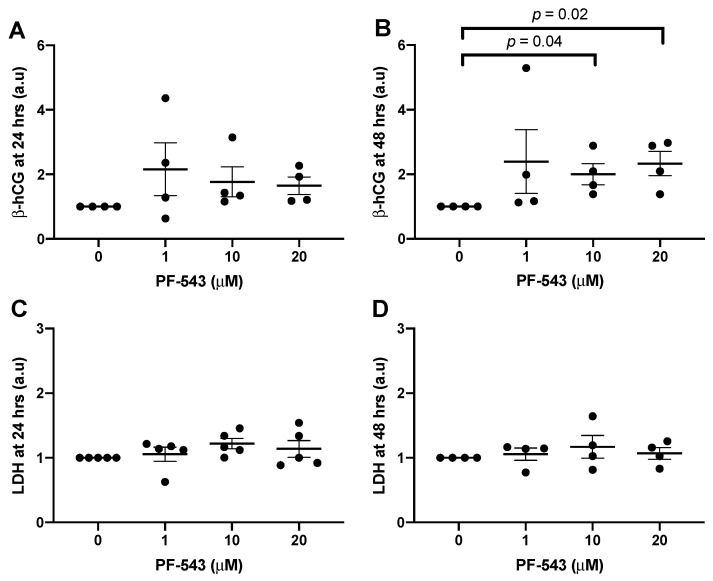
SphK1 inhibition with PF-543 increased β-hCG secretion after 48 h. Explants were pre-cultured for 4 days prior to treatment with a PF-543 dose response (0–20 μM). Supernatants were centrifuged at 15,000 RPM for 15 min prior to quantification of β-hCG and LDH. (**A**,**B**) represent β-hCG released into the supernatant after 24 and 48 h, respectively as measured by ELISA. (**C**,**D**) represent LDH released into the supernatant after 24 and 48 h, respectively as measured with a colorimetric assay. Results were normalized against total protein mass and normalized values were graphed as a ratio of change from the untreated control explant cultures. (*n* = 4–5, Mean ± SEM, Kruskal–Wallis test with the two-stage linear step-up procedure of Benjamini, Krieger, and Yekutieli post-hoc test).

The dosage of 1 μM PF-543 to inhibit SphK1 was established based on effective dosages in other cell types that examined different outcome measures. However, the effects of PF-543 on placental explant resyncytialization or cell death have not been investigated. We therefore wanted to determine whether higher doses of PF-543, that are still highly specific for inhibiting SphK1, would lead to enhanced effects. Thus, a PF-543 dose-response was performed. Explants were treated with either 0 μM, 1 μM, 10 μM, or 20 μM of PF-543 (*n* = 4) on day 4 of explant culture. β-hCG and LDH levels were measured. With respect to ST endocrine function, no significant changes were observed in β-hCG at any PF-543 concentration after 24 h of treatment (Figure 8A). However, inhibiting SphK1 activity with 10 or 20 μM of PF-543 for 48 h increased β-hCG secretion (*p* = 0.04, *p* = 0.02, respectively; Figure 8B). No significant changes in LDH levels were observed at any concentration of PF-543 at either time point (Figure 8C,D).

## 3. Discussion

The focus of this study was to determine whether the effects of elevated TNF-α, which induces a PE-like placental phenotype, characterized by poor ST function, high ST shedding, and high inflammation [12,13], are mediated through changes in SphK1 activity. S1P [28,37], which is produced by SphK1, and TNF-α [7,11,46] are already each independently associated with PE pathophysiology by inhibiting ST formation [12,39,40]. TNF-α increases SphK1 activation in HUVECs [25,26], hepatocytes [32], or a lung fibroblast cell line [33]. In the current study, TNF-α effects on syncytial hormone production and CT fusion were found to be independent of SphK1 activation. However, placental cell death and syncytial shedding induced by TNF-α were dependent on SphK1 activation. TNF-α also increased the release of 16 pro-inflammatory cytokines and 9 growth factors from cultured placental explants. However, TNF-α-induced changes were dependent on SphK1 activation for only five of these factors: IFNα2, IP-10, FGF2, and PDGF-AA were decreased, while IL-10 was increased after inhibiting SphK1 in the presence of TNF-α. Overall, we show that elevated TNF-α induced a PE-like placental phenotype that was mediated through SphK1 activation.

S1P, the product of SphK1, is elevated in the plasma of mothers with PE [37]. However, qRT-PCR and western blot data indicate that SphK1 mRNA and protein levels decrease in placental explants from women with PE compared to women with normal pregnancies [47]. Using immunofluorescence, we observed that SphK1 levels were higher overall in placental sections from PE compared to normal pregnancies. This discrepancy could be attributed to distinct epitopes recognized by different antibodies using different methods. However, we confirmed our staining results using qRT-PCR. Recently, however, an article by Liao et al. found a lower level of SphK1 protein levels in placental villi from PE pregnancies, also measured by immunofluorescence [48]. While patient characteristics were comparable to those in our study, this published study used paraformaldehyde fixation, that can reduce the fluorescent signal observed and result in poor preservation of the cytoplasm [49] and most likely the villous core. Indeed, our staining showed a strong signal for SphK1 in the villous core, whereas the images from Liao et al. show almost no expression aside from the outermost trophoblast layers. Liao et al. also show that placental SphK1 activity is decreased in the PE group; however, the method utilized does not discriminate between SphK1 and SphK2 [50]. Although S1P is elevated in the plasma of women with PE [37,38], it is unknown whether the placenta is the source of this surge. Liao et al. show that placental S1P levels are lower in mothers with PE [48] and did not measure circulating S1P in the mother. Since S1P is constantly transported to the extracellular space, it is unclear whether this decrease is due to increased transport, decreased synthesis or increased S1P lyase or lipid phosphatase activities.

The clinical characteristics of our samples indicated that women in the PE group had newborns with a lower gestational age than those in the healthy control group. However, it is unlikely that differences in SphK1 expression can be explained by gestational age differences. In our latest review, we note that circulating S1P levels remain constant throughout normal pregnancy and are therefore independent of gestational age [28]. Our previous studies also show that SphK1 levels in the decidua are higher with increasing gestational age [51]. For these reasons, we believe it is unlikely that higher levels of SphK1 in the placentas from mothers with PE are due to lower gestational ages.

TNF-α acts through two receptors: TNFR1 and 2 [7]. The signaling pathway through TNFR1 leads to cytotoxic effects in trophoblasts [52,53]. Recently, both S1P and SphK1 were deemed essential to the signaling cascade of TNF-α through TNFR1 in HEK293T and HeLa cell lines [24]. S1P and SphK1 act as cofactors for TNF-α receptor 1 signaling by interacting with TNF receptor-associated factor 1 [24] and ultimately leading to NF-κB activation [54]. However, there is a knowledge gap in understanding the relationship between TNF-α and SphK1 signaling in inducing placental malfunction. 

Hence, we aimed to decipher the independent role of SphK1 activation and its impact on TNF-α signaling in the placenta. Based on the existing knowledge regarding the role of SphK1 as an essential factor for TNF-α signaling [27], it was expected that inhibiting SphK1 would inhibit all effects of TNF-α. In our study, however, we found that some effects of TNF-α on placental function were independent of SphK1 activation. Although TNF-α decreased resyncytialization of the explants as expected, this was not changed when SphK1 activity was inhibited during treatment. This implies that TNF-α hinderance of trophoblast fusion is independent of SphK1 activation. 

On the other hand, only inhibiting SphK1 with PF-543 hindered fusion as well, suggesting an important role for this kinase and its product, S1P in the fusion process. This is contrary to what Singh et al. showed using DMS (N,N-Dimethyl-D-erythro-sphingosine) to inhibit SphK1 [55]. One explanation for this disparity is that DMS has many off-target effects especially on trophoblast metabolism [40]. Additionally, DMS inhibits both SphK1 and SphK2 and is a protein kinase C inhibitor, which is downstream of S1PR2-3 signaling [56]. PF-543, however, is reported to be specific and efficient, inhibiting over 95% of SphK1 activity at 1 μM with a 132-fold selectivity for the SphK1 isoform over SphK2 [57]. Moreover, our experiments were done in chorionic explant cultures where CTs are in context with the surrounding tissue and extracellular matrix as opposed to those done by Singh et al. which used cultured primary trophoblasts only. Singh and colleagues show that SphK1 levels decrease as BeWo choriocarcinoma cells fuse into a multinucleated ST [40]. This could imply the importance of SphK1 in inducing CT fusion and its downregulation once fusion is complete.

In contrast to the important role SphK1 may play in CT fusion, inhibiting SphK1 activity alone increased β-hCG release from cultured placental explants. The seemingly contradictory effects of SphK1 inhibition on syncytial function versus CT fusion in our findings might point to differential effects of SphK1 inhibition depending on the cell type, CT versus ST. This, however, remains to be investigated. While CT fusion and β-hCG release are both used as markers for syncytial differentiation, it is important to remember that these processes occur by separate signaling pathways and in different cell types. A differential effect on fusion versus endocrine function was also seen by Singh et al. when using the non-specific DMS to inhibit SphK; however, the results were opposite to ours, with increased CT fusion and decreased β-hCG release.

Interestingly, in our study, the effects on β-hCG release were only observed with 10 ng/mL of TNF-α, but not with 1 ng/mL of TNF-α. TNF-α was previously established to have pro-apoptotic effects in the syncytium and to decrease β-hCG release at 10 ng/mL in villous explants [12], whereas 1 ng/mL of TNF-α had no effect on β-hCG release [12]. Thus, our results are in agreement with this previous literature. TNF-α at the lower concentration of 1 ng/mL did, however, decrease trophoblast fusion. This decrease, as mentioned, had no consequences on β-hCG release. This could have occurred because minimal ST could be sufficient to produce β-hCG at healthy levels even in the presence of 1 ng/mL of TNF-α; however, 10 ng/mL of TNF-α overcomes the compensating response triggered by ST.

The syncytium is maintained by a balance of trophoblast fusion/differentiation and syncytial shedding. Since cell damage and syncytial shedding are increased in PE, we assessed the levels of LDH release and PLAP-positive shed particles, respectively. Inhibiting SphK1 alone had no effect on placental cell membrane damage or syncytial shedding. However, these measures were increased in explants treated with TNF-α alone, reflecting increased cell death. Inhibiting SphK1 while treating with TNF-α decreased cell death and syncytial shedding induced by TNF-α alone, indicating that TNF-α exerts its effects on cell death and shedding through SphK1. LDH is a cytosolic enzyme that can induce autophagy in tumor cells [58]. TNF-α [59], as well as SphK1 activation [60], increases autophagy. In fact, ceramide, a precursor of S1P, increases autophagy in a trophoblast cell line [61]. Autophagy is increased in the placental villi of women with PE [59], but the regulators of this process in the placenta are still unclear. Our results show that TNF-α-induced LDH release is dependent on SphK1 activity. Further research would investigate whether the TNF-α-SphK1-LDH release pathway regulates autophagy in the placenta. 

Studies in epithelial and endothelial cells show TNF-α administration increases activity and expression of SphK1 levels [24,25,26,27]. Indeed, our study showed that TNF-α is dependent on SphK1 activity to induce cell death and syncytial shedding in the placenta. Potentially, this could occur through TNF-α-induced activation of NF-κB-mediated cell death pathways [24]. NF-κB is a known activator of cell fate pathways as well as pro-inflammatory and growth factor production. Thus, SphK1-mediated TNF-α induction of IFNα2, IP-10, FGF2, and PDGF-AA release could, in fact, be occurring due to NF-κB activation. While the role of IFNα2 is not yet clear in the placenta, this type I interferon is expressed by epithelial cells and is responsible for upregulating genes of proteins involved in immune responses in the bovine endometrium [62]. IFNα2 also increases choriocarcinoma trophoblast cell resistance to methotrexate by regulating cell viability [63]. On the other hand, IP-10 inhibits angiogenesis by reducing endothelial cell viability and proliferation in various cell types, including human vascular endothelial cells. IP-10, however, also stimulates vascular smooth muscle cell motility and therefore differentiation [64,65]. This step is critical for spiral artery remodeling in early pregnancy. Improper spiral artery remodeling is strongly associated with the development of PE. FGF2 and PDGF-AA are also upregulated in response to cell damage [66,67] as a compensatory response. In particular, PDGF-AA rescues trophoblasts from TNF-α-induced cell death [30]. Thus, this interaction might occur downstream of the cell death pathway induced by TNF-α, which is dependent on SphK1 activation. 

In our study, the only factor that TNF-α treatment of placental explants decreased was IL-10 and inhibiting SphK1 activation not only reversed this effect but also led to increased levels above the control. Siwetz et al. show that IL-10 levels are unchanged in response to TNF-α in first-trimester villous explants [13]. However, IL-10 circulating levels are lower in PE in second- and third-trimester pregnancies [68]. This supports the idea that high levels of TNF-α in PE could be the reason behind the drop in circulating IL-10 levels. The TNF-α-induced decrease in IL-10 release was dependent on SphK1 activation. As summarized by Kalkunte et al., IL-10 is an anti-inflammatory cytokine that is crucial for pregnancy maintenance [69]. Its levels are normally decreased at term to allow the rise in inflammation necessary for the induction of labor. Hence, high levels of TNF-α lead to a further decrease in IL-10, which would skew the balance towards a hyperinflammatory state. IL-10 serves a protective role in pregnant women exposed to hypoxia, a characteristic of PE. Pregnant IL-10 knockout mice exposed to a hypoxic environment developed placental damage and PE symptoms. Treating these mice with recombinant IL-10 reversed the PE symptoms induced by hypoxia. IL-10 is also protective in other aspects of the disorder, such as inflammation and vascular function, which are all summarized in the following review [69]. Interestingly, IL-10 acts as a strong protector against autophagic activity [59]. Hence, the SphK1-dependent TNF-α decrease of IL-10 could be a potential mechanism of TNF-α-induced autophagy in the placenta.

TNF-α induced the release of a wide variety of inflammatory and growth cytokines but the majority of these were independent of SphK1 activation. This could be because TNF-α acts on specific cell types in the placenta that release some cytokines and not others. Hence, TNF-α could be dependent on SphK1 activation only in specific cell types. Alternatively, TNF-α could be inducing certain cytokines through TNFR1, and some through TNFR2 whose signaling and interaction with SphK1 have yet to be investigated. Interestingly, inhibiting SphK1 led to a decrease in TNF-α release, implying that SphK1 activity induces TNF-α release from the placenta. This release could be a normal physiological response when SphK1 activation levels are within normal ranges and could lead to the release of TNF-α to increase normal processes like angiogenesis and proliferation [7]. However, high levels of TNF-α in pathological conditions such as PE could contribute to high levels of SphK1 activation in the placenta, which would further amplify TNF-α levels and exacerbate the PE-like phenotype, suggesting a feed-forward loop. The role of SphK1 activity in mediating the TNF-α-induced decrease in IL-10 as well as the decrease in TNF-α itself has important translational implications in other inflammatory disorders, particularly those associated with autoimmune diseases such as inflammatory bowel disease [70]. Our findings propose investigating whether SphK1 inhibitors or agonists/antagonists regulating S1P signaling in general could be synergistic with TNF-α-targeted antibodies for treatment of these disorders.

Finally, only GRO-α increased in response to SphK1 inhibition. While the role of GRO-α in placental and trophoblast function throughout pregnancy has not been extensively investigated, GRO-α is commonly secreted from trophoblasts in response to cell damage [71]. In our study, inhibiting SphK1 could have led to a specific damage-induced signaling pathway which was not triggered by TNF-α and which led to the increase in this cytokine.

Explants are a complex three-dimensional culture with multiple cell types, matrices, and cell-cell interactions. Hence, potent inhibitors like PF-543 can potentially bind proteins, be metabolized, or sequester within the tissue, thereby losing some potency. To investigate if increasing concentrations of PF-543 could have distinguishable effects on explant syncytialization, we exposed explants to increasing PF-543 concentrations that were still within the specific range [57]. PF-543 at 10 and 20 μM increased the release of β-hCG from villous explants without affecting cell viability. Schnute et al. shows that 10 μM of PF-543 in a monolayer cell culture does not bind any off-target kinases from the kinase profiling panel [57]. Moreover, it has a 132-fold selectivity for SphK1 over SphK2 [57]. As mentioned, PF-543 has not been tested in trophoblasts or in placental explants previously. Nonetheless, 1 μM [72,73] and even 10 μM [74] of PF-543 is a commonly used concentration in a monolayer culture of cells isolated from other tissues.

Certain studies show a difference in function for S1P produced by SphK1 or SphK2 [75]. SphK1 is the cytoplasmic isoform which contributes mainly to extracellular S1P that signals through its receptors. SphK2 is located in the nucleus and the rough endoplasmic reticulum and is the main producer of S1P that signals intracellularly [76]. SphK2 is poorly studied in the placenta and its interaction with TNF-α on placental function is unknown. 

Currently, there are no definitive treatments for PE except for delivery of the fetus, which often leads to preterm birth [77]. This study showed that TNF-α induced its effects on several aspects of placental function by activating SphK1 and identifies the S1P signaling pathway as a potential treatable target. However, since blocking SphK1 activity also inhibited trophoblast fusion [55] and since S1P has many diverse protective functions, the market-available SphK1 inhibitors [78] may pose off-target effects that will impact placental health and potentially fetal health through placental programming. Moreover, inhibition of SphK1 activity will reduce the synthesis of S1P which is protective and anti-apoptotic at normal levels [28]. One way to mitigate this is to target specific S1P receptors. For instance, S1PR1 is a key player in counteracting TNF-α induced release of MCP-1 and IL-8 in placental vascular tissues [28]. S1PR2 activation induces IL-6 secretion from trophoblasts [28,75]. Further studies to understand the role of inflammation and its impact on S1P signaling in syncytial development and function are crucial to better understand the mechanisms leading to PE and the development of novel therapeutics to treat this pregnancy disorder.

## 4. Materials and Methods

### 4.1. Placenta Samples

Women were recruited and provided written informed consent. The study was approved by the University of Alberta Health Research Ethics Board (Pro00034274, continuously approved since 15 November 2012 with the most recent renewal approved 2 January 2022) and conformed to the standards set by the Declaration of Helsinki. Term placentae were collected immediately after elective caesarean section deliveries at the Royal Alexandra Hospital, Edmonton, AB, Canada, from women with no health complications and from women with PE. Women with PE were defined as those who had blood pressures above 140/90 mmHg on two occasions measured 6 h apart and at least 30 mg/dL proteinuria in a 24 h collection. Seventeen women with healthy pregnancies and 12 women with PE were enrolled in the study. Placental biopsies from all women were collected for staining. Biopsies from 13 placentas from the healthy control group and nine placentas from the PE group were collected and processed for qRT-PCR. Table 1 displays clinical characteristics of all 17 women with healthy pregnancies and nine women with PE enrolled in the study.

### 4.2. SphK1 Immunohistochemistry of Placental Sections

Placental biopsies were collected from placentas within 2 h of delivery from the area between the umbilical cord and the placental edge. Three evenly spaced 6 mm^2^ biopsies from each patient were embedded into one mold with Tissue-Tek^Ⓡ^ O.C.T. (#4583, ThermoFisher Scientific, Waltham, MA, USA). Three 8 μm sections from varying depths of one mold were cut on a cryostat and these sections were placed on a Superfrost slide (ThermoFisher Scientific, Waltham, MA, USA). The sections were fixed with 100% ice-cold methanol for 20 min in −20 °C and blocked for 1 h with 10% normal goat serum. The sections were then incubated in a moist chamber simultaneously with a rabbit anti-SphK1 primary antibody (1:80, ab46719, Abcam, Cambridge, UK) and a mouse anti-E-cadherin primary antibody (1:200, 55413, R&D Systems, Minneapolis, MN, USA) in 1× PBS overnight. Slides were washed 3 times with 1× PBS for two minutes per wash. AlexaFluor goat anti-rabbit 488 and goat anti-mouse 594 (1:200, A32731 and A11032, Invitrogen, Waltham, MA, USA) secondary antibodies were added simultaneously and incubated in the dark for 1 h at room temperature. After washing 3 times with 1× PBS, the sections were incubated for 10 min in the dark with DAPI (4′,6-diamidino-2-phenylindole, 1:100, D1306, Invitrogen, Waltham, MA, USA). The slides were washed again and mounted with PermaFluor Aqueous Mounting Medium (TA-030-FM, ThermoFisher Scientific, Waltham, MA, USA). Three randomly selected areas from each section were imaged using the 20× objective on Zeiss LSM 700 microscope and Zen Black software (Zeiss, Toronto, ON, Canada). Images were analyzed using ImageJ software (Version 1.52a, U.S. National Institutes of Health, Bethesda, MD, USA). Briefly, images were set to a consistent threshold value, and mean integrated density was calculated. Integrated density values were normalized to the number of nuclei in the image, which were calculated through the “Analyze particles” function on ImageJ. Normalized values for each sample were averaged and the standard error of the mean was determined.

### 4.3. RNA Isolation

Three evenly spaced 6 mm^2^ placental biopsies were obtained from term placentas in the area between the umbilical cord and the edge of the placenta. Samples were collected within 2 h of delivery and biopsies from each placenta were placed together in a single tube. Placental biopsy tissues were placed into liquid nitrogen. The frozen samples were wrapped with aluminum foil, re-submerged in liquid nitrogen, and crushed using a mortar and pestle until the tissues were in powder form. Next, each sample was transferred into a 2 mL PCR tube containing a single steel bead (QIAGEN, Toronto, ON, Canada). Each tube was then filled with 1 mL of TRIzol reagent (Life Technologies, ThermoFisher Scientific, Waltham, MA, USA). The samples were lysed using a TissueLyser II (QIAGEN, Toronto, ON, Canada) at a setting of 25.00 frequency/s for 2 min, repeated 3 times. 200 μL of chloroform was then added to each sample before vigorously shaking for 1 min, followed by a 5 min incubation at room temperature. Samples were then centrifuged for 15 min at 15,000 rcf at 4 °C. Following this step, the RNA layer (aqueous top layer) was extracted and transferred to a new 2 mL tube and the Invitrogen RNA isolation kit (AM1912, Toronto, ON, Canada) was used to purify the isolated RNA. Finally, quantification of the RNA in these samples was carried out using the Nanodrop Spectrophotometer (ThermoFisher Scientific, Waltham, MA, USA). Samples that were below a 260/280 ratio of 1.7 were discarded, but this was very uncommon with the isolation kit that was used. 

### 4.4. Quantitative RT-PCR

Purified RNA was reverse transcribed into cDNA using 500 ng of total RNA, 4 μL of ABM 5× all-in-one RT MasterMix (AT6490, ABM Life Sciences, Vancouver, BC, Canada), and UltraPure TMDistilled Water (110977-015, Invitrogen, Waltham, MA, USA) as needed to reach a final volume of 20 μL. The primer sequences for SphK1 and the housekeeping gene, hypoxanthine-guanine phosphoribosyl transferase (HPRT1), were designed using OligoPerfect Designer (Integrated DNA Technologies, Coralville, IA, USA). SphK1 forward: 5′-CTG GCA GCT TCC TTG AAC CAT3′; SphK1 reverse: 5′-TGT GCA GAG ACA GCA GGT TCA-3′; HPRT1 forward: 5′-GAC CAG TCA ACA GGG GAC ATA A-3′; HPRT1 reverse: 5′-AAG CTT GCG ACC TTG ACC-3′. The PCR reaction was carried out using Applied Biosystems Power Up TM SYBR^®^ Green MasterMix (100029284, Life Technologies, ThermoFisher Scientific, Waltham, MA, USA), 0.6 μL of primers (10 μM), 6.3 μL of UltraPure TM Distilled Water, and 2.5 μL of cDNA from each sample. The ΔΔCT method of relative quantification was used to analyze the results [79].

### 4.5. Placental Explant Culture

Placental chorionic villi were dissected within 1–2 h following delivery and washed 3 times with gentle shaking using Hanks’ Balanced Salt Solution (Hyclone Laboratories Inc, ThermoFisher Scientific, Waltham, MA, USA). A self-regenerating explant culture method was adapted from Siman et al. and Trowell [80,81] by culturing explants at the gas-liquid interface. Briefly, three 2 mm blocks of chorionic villi were placed onto inserts with 8 μm pore polyethylene terephthalate membranes (353182, ThermoFisher Scientific, Waltham, MA, USA). Inserts were placed into wells in a companion 12-well plate. 550 µL of Iscove’s Modified Dulbecco’s Media (IMDM, Hyclone Laboratories Inc, ThermoFisher Scientific, Waltham, MA, USA) with 1% Penn/Strep, 1% antibacterial/antimycotic, and 1% ITS + 1 Liquid Media Supplement (MilliporeSigma, Toronto, ON, Canada) was added to the wells in the companion plates. The plates were incubated at 37°C in 5% CO_2_ and the media was changed every 48 h. 

### 4.6. Treatments and Experimental Protocols 

Explants were incubated for 4 days to allow the shedding of the original syncytium [82] and all experimental treatments began on day 4 of culture at the start of the ST regeneration phase. Explants were treated with 0–20 µM PF-543 (PZ0234, MilliporeSigma, Oakville, ON, Canada), a specific inhibitor of SphK1 [57]. Explants were also treated with TNF-α (1 or 10 ng/mL, a kind gift from Hoffmann-La Roche, Basal, Switzerland) alone. To test if inhibition of SphK1 could impact TNF-α effects, explants were pretreated with PF-543 (1 μM) for 30 min before TNF-α (1 or 10 ng/mL) administration. Treatments were done in duplicates for 24 or 48 h. 

### 4.7. Trophoblast Fusion and E-Cadherin Staining 

Cultured explants from duplicate wells (3 explants/well) were fixed in 1% paraformaldehyde in PBS, embedded and sectioned as described above. The 3 villi from each well were placed in 1 mold that was sectioned at 3 varying depths and these sections were placed onto 1 slide. Similar blocking and washing procedures were followed as described above. Samples were then incubated with the E-cadherin primary antibody (1:200) overnight. AlexaFluor-488 goat anti-mouse secondary antibody (1:200) and DAPI (1:100) were used as described above. Slides were similarly mounted as before and 20 Z-stacks of each image were captured with a confocal Zeiss LSM 700 microscope and Zen Black software (Zeiss, Toronto, ON, Canada). Three randomly selected fluorescent images were captured per each section with the 20× objective and analyzed using ImageJ software Version 1.52a. E-cadherin localizes at the apical membrane of the CT layer, which fuses into the basal membrane of the syncytial region. Syncytialized portions were defined as nuclei external to the E-cadherin demarcation. The total area of the syncytialized portions of the explants were measured and normalized against the total surface area of the villi. 

### 4.8. BCA Protein Assay 

Explant tissues were placed in lysis buffer (20 mM Tris, 5 mM EDTA, 10 mM Na_4_P_2_O_7_, 100 mM NaF, 1% NP-40) with protease inhibitor cocktail (Sigma-Aldrich, St. Louis, MO, USA) mixed in a 100:1 ratio in a tube with a metal bead. Samples were lysed using the Qiagen Tissue Lyser III (Qiagen, Hilden, Germany) through three cycles of 25 shakes/s, with 2 min per cycle. Total protein mass was quantified using the Pierce BCA Protein Assay Kit (ThermoFisher Scientific, Waltham, MA, USA) with an albumin standard following manufacturer recommendations. Samples were run in duplicates at a 1:10 dilution. Absorbance was measured with a Synergy HTX spectrophotometer (Agilent Technologies, Santa Clara, CA, USA) at a 562 nm wavelength.

### 4.9. Lactate Dehydrogenase (LDH) Quantification 

Lactate dehydrogenase (LDH) is released from all cell types as a result of cell membrane damage and is a marker for cell death. Experimental supernatants were centrifuged at 15,000 rpm for 15 min, and supernatant LDH levels were measured with the CytoTox 96^®^ Non-Radioactive Cytotoxicity Assay following the manufacturer’s protocol (Promega, Madison, WI, USA). If LDH were present, it would result in a color change to red due to the formation of Formazan, and the absorbance was read at 562 nm using a Synergy HTX spectrophotometer. Samples were run at a 1:2 dilution in duplicates. Culture media alone was used as a negative control and subtracted from the experimental sample absorbance values. Values were normalized to total protein mass and calculated as a ratio of the no-treatment control. Duplicate wells for each treatment were averaged.

### 4.10. Quantification of the Beta-Subunit of Human Chorionic Gonadotropin (β-hCG) 

β-hCG is secreted by the ST and is a marker of a functional ST. β-hCG from experimental supernatants was measured using an enzyme-linked immunosorbent assay (ELISA) with the ELISA 1911 kit (DRG Diagnostics, Marburg, Germany) following the manufacturer’s instructions. After centrifuging the samples at 15,000 rpm for 15 min, the supernatant was collected and run at a 1:5 dilution with the manufacturer’s supplied buffer. Absorbance was measured with the Synergy HTX spectrophotometer at a wavelength of 450 nm. The values from each assay obtained from culture medium alone were subtracted from experimental sample values. β-hCG concentrations were extrapolated using spectrophotometer measurements compared to those from a standard curve. Measured concentrations were normalized to total protein mass per well and calculated as a ratio of the no-treatment control. Duplicate wells for each treatment were averaged.

### 4.11. Quantification of Human Placental Alkaline Phosphatase Activity (PLAP)

PLAP activity was measured from villous particles shed into the bottom wells of the insert cultures to estimate the amount of ST shedding in response to various treatments. All particles reaching the bottom well were smaller than the 8 μM pore size. Experimental supernatants were diluted 10 times in manufacturer supplied buffer and analyzed following the manufacturer’s protocol (ab83369, Abcam, Cambridge, UK). Briefly, phosphorylated p-Nitrophenol was added to the samples as a substrate. The alkaline phosphatase in the samples converts the substrate into the non-phosphorylated p-Nitrophenol, ultimately leading to a change in color. Absorbance was measured with a Synergy HTX spectrophotometer at a 405 nm wavelength. The values from each assay obtained from culture medium alone were subtracted from experimental sample values. PLAP activity of samples was extrapolated using spectrophotometer measurements compared to those from a standard curve. Measured concentrations were normalized to total protein mass per well and calculated as a ratio of the no-treatment control. Duplicate wells for each treatment were averaged.

### 4.12. Quantification of Cytokines 

Supernatants from cultured explants were tested for 42 cytokines/chemokines in duplicate: EGF, Eotaxin-1, FGF-2, Flt-3L, CX3CL1, G-CSF, GM-CSF, GRO-α, IFN-α2, IFN-γ, IL-1α, IL-1β, IL-1ra, IL-2, IL-3, IL-4, IL-5, IL-6, IL-7, IL-8, IL-9, IL-10, IL-12(p40), IL-12(p70), IL-13, IL-15, IL-17A, IL-18, IP-10, MCP-1, MCP-3, MDC, MIP-1α, MIP-1β, PDGF-AA, PDGF-AB/BB, RANTES, sCD40L, TGF-α, TNF-β, VEGF-A. Tests were performed by Eve Technologies (Calgary, AB, Canada) using a Luminex array with a Bio-Plex™ 200 system (Bio-Rad Laboratories Inc., Hercules, CA, USA) and a (HD42) kit (Millipore, St. Charles, MO, USA).

### 4.13. Statistics and Analysis 

Results were analyzed with GraphPad Prism (GraphPad Software, San Diego, CA, USA). Results from imaging were analyzed through Students’ t-test. Normalized fusion, β-hCG, LDH, PLAP, and cytokine/chemokine results from cultured explant experiments were analyzed by two-way ANOVA and two-stage linear step-up procedure of Benjamini, Krieger, and Yekutieli post-hoc test to control for the false discovery rate. Dose-response experiments were analyzed by non-parametric Kruskal–Wallis test and the two-stage linear step-up procedure of Benjamini, Krieger, and Yekutieli post-hoc. A *p* value less than 0.05 was considered significant. All groups were subjected to post-hoc analyses. Only significant post-hoc *p*-values are shown in the figures.

## Figures and Tables

**Table 1:** Clinical Characteristics of Pregnancies. Samples were analyzed with a Student’s *t*-test or Mann–Whitney test when non-parametric (Mean ± SEM).

Pregnancy	Gestational Age (Weeks)	Infant Weight (g)	Systolic Blood Pressure (mmHg)	Diastolic Blood Pressure (mmHg)	Percent Positive for Proteinuria (%)
Normal (*n* = 17)	38.9 ± 0.3	3074 ± 86.8	113.8 ± 3.7	71.4 ± 3.6	0
Preeclampsia (*n* = 12)	37.2 ± 0.3	2967 ± 274	155.3 ± 7.6	107.9 ± 15.6	100
*p*-value	0.003	ns	*p* < 0.0001	0.008	N/A

## Data Availability

All data is reported as results in the manuscript or in the supplementary figures or table. Any other data will be shared by reasonable requests made to the corresponding author.

## Supplementary Materials

The following supporting information can be downloaded at: https://www.mdpi.com/article/10.3390/ijms23073750/s1.
