# Supplementary material for: Tumor Necrosis Factor-α Induces a Preeclamptic-like Phenotype in Placental Villi via Sphingosine Kinase 1 Activation
Source: Int J Mol Sci. 2022 Mar 29;23(7):3750. doi: 10.3390/ijms23073750 (PMC8998215; doi:10.3390/ijms23073750)
Supplement: Supplementary file 1 [file ijms-23-03750-s001.zip › ijms-1603803-supplementary.pdf]

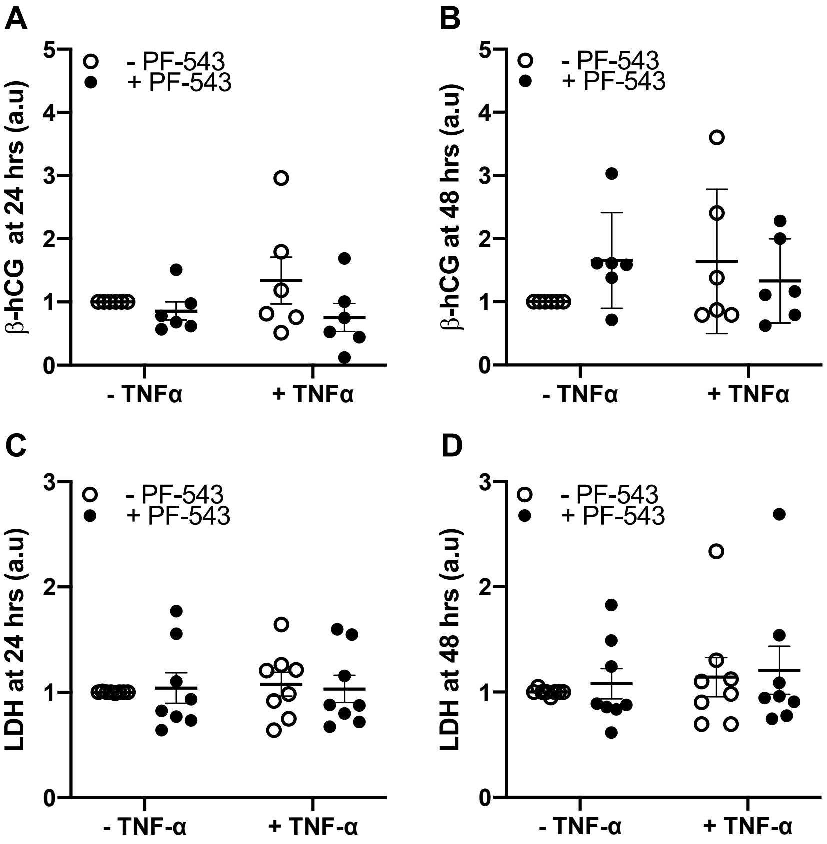

Figure S1: No effects of 1 ng/mL of TNF*α* with or without 1 μM PF-543 on β-hCG or LDH release into supernatants at 24 or 48 hrs. Explants were pre-cultured for 4 days prior to treatment with 1 ng/mL of TNF-α, 1 μM PF-543, a co-treatment of both or a no treatment control for 24 or 48 hours. Supernatants were centrifuged at 15000 RPM for 15 mins prior to quantification of β-hCG and LDH. (A) and (B) represent β-hCG released into supernatants after 24 and 48 hours, respectively as measured by ELISA (n=6). (C) and (D) represent LDH released into supernatants after 24 and 48 hours, respectively as measured with a colorimetric assay (n=8). Results were normalized against total protein mass and normalized values were graphed as a ratio of change from the untreated control explant cultures. Results were analyzed by Two-Way ANOVA with the Two-stage linear step-up procedure of Benjamini, Krieger, and Yekutieli post-hoc test. (Mean ± SEM)

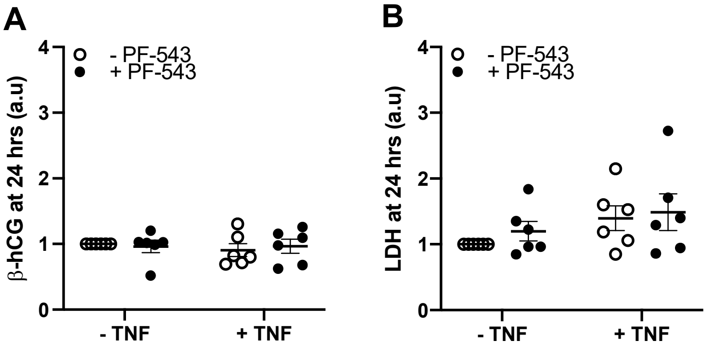

Figure S2: No effects of 10 ng/mL of TNF-α with or without inhibition of SphK1 on LDH release or β-hCG secretion into supernatants at 24 hrs. Explants were pre-cultured for 4 days prior to treatment with 10 ng/mL of TNF-α, 1 μM PF-543, a co-treatment of both or a no treatment control for 24 hours. Supernatants were centrifuged at 15000 RPM for 15 mins prior to analysis. (A) and (B) represent β-hCG release and LDH released into the supernatant after hours, respectively. Results were normalized against total protein mass, and normalized values were graphed as a ratio of change from the untreated control explant cultures. Results were analyzed by Two-Way ANOVA with the Two-stage linear step-up procedure of Benjamini, Krieger, and Yekutieli post-hoc test. (n=5, Mean ± SEM).

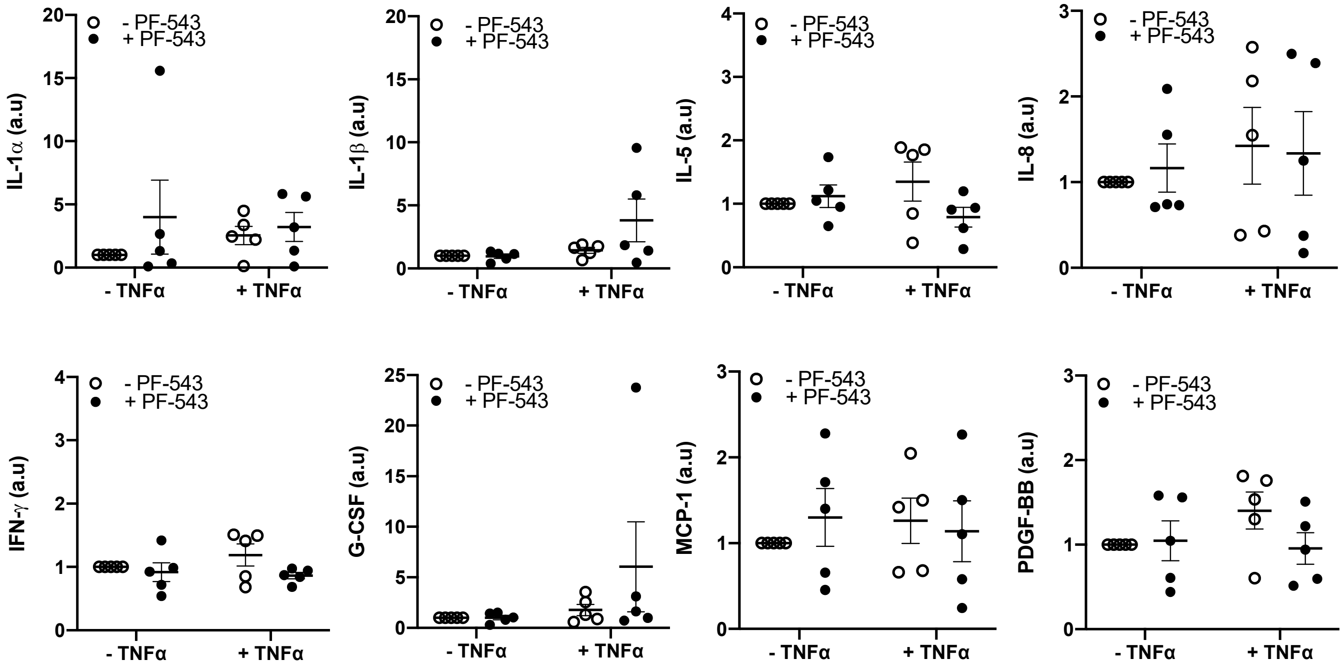

Figure S3: Inhibiting SphK1 with PF-543 and/or TNF-α treatment had no effect on the release of some cytokines, factors, or chemokines. Explants were pre-cultured for 4 days prior to treatment with 10 ng/mL of TNF-α, 1 μM PF-543, a co-treatment of both or a no treatment control for 48 hours. Supernatants were centrifuged at 15000 RPM for 15 mins prior to quantification of inflammatory cytokines, growth factors, and chemokines on a multiplex array. Results were normalized against total protein concentration, and normalized values were graphed as a ratio of change from the untreated control explant cultures. Results were analyzed by Two-Way ANOVA with the Two-stage linear step-up procedure of Benjamini, Krieger, and Yekutieli post-hoc test. (n=5, Mean ± SEM)
